# Including people with lived experience in the development of the self-Dehumanisation in Psychosis Scale (DiPS): a reflective account

**DOI:** 10.1186/s41687-025-00981-3

**Published:** 2025-12-10

**Authors:** Tom A. Jenkins, Anneli Bale, Linda Alush, Ed Brooks, Ian Carter, Eva Roberts, Pamela Jacobsen, Paul Chadwick

**Affiliations:** 1 Department of Psychology, University of Bath, Claverton Down, Bath, BA2 7AY UK; 2 School of Health and Social Wellbeing, University of the West of England, Frenchay Campus, Bristol, BS16 1QY UK; 3 Bristol, UK

## Abstract

**Background:**

Lived experience involvement in measure development is recognised as best practice. This article is a reflective account, co-written between researchers and PPIE (Patient and Public Involvement and Engagement) consultants with lived experience of psychosis, about the impact of lived experience involvement in developing the self-Dehumanisation in Psychosis Scale (DiPS). Reporting is guided by the Guidance for Reporting Involvement of Patients and the Public 2 – Short Form (GRIPP2-SF).

**Methodology:**

In developing the DiPS, people with lived experience of psychosis contributed both as research participants and as PPIE consultants across multiple stages of development. This included in study document review; identification of domains; item generation, refinement, and amendment; psychometric validation; and dissemination.

**Results:**

Contributions made by people with lived experience were significant across all stages of developing the DiPS, including developing and shaping conceptual understanding of self-dehumanisation; item generation and selection; and improving the comprehensibility and acceptability of the measure.

**Discussion:**

Working collaboratively with people lived experience in measure development can be of benefit both to the validity of the measure, and to those involved. We reflect on the value and importance of working collaboratively, and offer recommendations based on our experiences for researchers co-developing measures.

## Background

Outcome measures in health research have historically been developed without involvement of people with lived experience [1], leading to concerns they measure clinician’s perception of a problem, rather than the person’s [2]. There are at least two significant issues with this. Firstly, content validity of the measure may be impaired, meaning the measure is less likely to capture the outcome as experienced by the target population [3, 4]. Secondly, omission of people with lived experience in developing outcome measures prohibits them from contributing towards a shared societal understanding of their experience, a form of hermeneutical injustice [5].

In a study comparing the psychometric properties of a service user-developed outcome measure with a conventionally generated outcome measure, Bakolis and colleagues [6] measured inpatient service user’s perceptions following a staff training intervention. They found that the Views on Inpatient Care (VOICE) [7], developed by service users, demonstrated a greater sensitivity to change than the Service Satisfaction Scale: Residential Services Evaluation (SSS-Res) [8], developed without service users, despite both measures having good psychometric properties. Not only was this measure better able to measure therapeutic benefit to service users, but the outcome itself was most relevant to that valued by service users. In psychosis research, the Choice of Outcome In Cbt for psychosEs (CHOICE) [9], Questionnaire about the Process of Recovery (QPR) [10], and Semi-structured Interview Measure of Stigma (SIMS) [11] also provide notable examples of where lived experience involvement has been used effectively to create measures that are more valid, meaningful, and acceptable to those who complete them. This ensures relevant outcomes are captured accurately, and can aid understanding and meaning-making of experience [12, 13].

Frameworks developed by Rose and colleagues [14] and Carlton and colleagues [15] describe meaningful co-production of outcome measures, with power shared between researchers and PPIE collaborators across multiple stages of development. However, implementation of these frameworks remain limited, with a scoping review reporting that only 6.7% of 193 patient reported outcome measures involved patients at all stages of development [1], and a systematic review of measures of anxiety for people with psychosis found that none out of eleven measures had any input from people with lived experience [16]. This shows a clear gap between best practice guidance, and the current evidence base, highlighting a need for more lived experience involvement in measure development.

### Self-Dehumanisation in Psychosis Scale (DiPS)

Self-dehumanisation is when a person feels less or other than human. Originally studied in social psychology [17], there is growing clinical interest in self-dehumanisation as a transdiagnostic phenomenon, particularly in relation to psychosis, suicidality, and alcohol-use disorder [18]. A systematic review found none of 14 existing measures of self-dehumanisation included any lived experience involvement, nor were any developed specifically for psychosis [19]. It is for these reasons we developed the self-Dehumanisation in Psychosis Scale (DiPS), following best practice guidance for scale development from Boateng and colleagues [20]. Full development and validation of the DiPS is reported elsewhere [21].

### Aim

We aimed to work collaboratively with people with lived experience of psychosis to develop a new measure for self-dehumanisation in psychosis (DiPS). The purpose of this paper is to provide a reflective account of the process of lived experience involvement in development of the DiPS, as a case study exemplar, and highlight the impact people with lived experience of psychosis made on improving the measure. Involvement is reported in line with the Guidance for Reporting Involvement of Patients and the Public 2 – Short Form (GRIPP2) [22].

## Methods

### Lived experience involvement

People with lived experience of psychosis were involved both as research participants and as co-researchers during this research. For the purpose of this paper, we will refer to research participants as ‘participants’ and co-researchers as Patient and Public Involvement and Engagement (‘PPIE’) consultants. We will also use the term ‘lived experience involvement’ as an umbrella term, to refer to working with people with lived experience in either or both of these capacities.

Five PPIE consultants with personal experience of psychosis and mental health service use comprised the DiPS lived experience consultation group. PPIE consultants all expressed interest in involvement after initial contact was made through a newsletter sent to the PPIE committee at the University of Bath. All five members are co-authors on this paper.

Research participants were recruited via Hearing Voices Network, MQ Participate, and adverts posted in the University of Bath, with a separate recruitment window for each part of the study. An overview of lived experience involvement can be found in Table 1.

**Table 1:** Involvement of people with lived experience of psychosis in developing the DiPS as PPIE consultants and participants

Stage	PPIE consultants	Research participants
1. Study document review	Reviewed and provided input on participant-facing study documentation	–
2. Identification of domains	Focus group to discuss interview themes	Took part in qualitative interviews [23] to explore self-dehumanisation
3. Item generation, refinement, and amendment	Focus group to review and generate items	Items generated from qualitative interview transcripts [23] Participated in cognitive interviews and Delphi survey
4. Psychometric validation	Advised on item reduction and presentation	Completed the outcome measure and provided data for item analysis
5. Dissemination	Advised on dissemination strategies, co-designed dissemination materials, co-presented at conference	–

#### Study document review

PPIE consultants gave feedback on the content of all participant facing study documentation. This included participant information sheets, debrief forms, consent forms, and recruitment posters.

#### Identification of domains

An initial theoretical framework for self-dehumanisation in psychosis was developed drawing from qualitative research on experiences of self-dehumanisation in voice-hearers [23] and a literature review of dehumanisation theory. This framework was then presented to and discussed by the DiPS lived experience consultation group in a focus group to: (1) explore the domains and identify gaps and (2) gather views on the value and importance of a measure of self-dehumanisation in psychosis. Prior to the group meeting, the first author (TJ) shared a brief introduction to self-dehumanisation, and a summary of the proposed domains with PPIE consultants.

#### Item generation, refinement, and amendment

Items were generated from qualitative interview transcripts [23]; existing measures of self-dehumanisation; discussions from the first focus group; and discussions between the research team. The DiPS lived experience consultation group and first author (TJ) then met again in a second focus group to review these items and generate further items. Items were sent in advance of the meeting, then each discussed in turn over the course of the meeting. Items suggested during the meeting would then be discussed by the supervisory team before a shortlist was finalised.

This shortlist was then presented to research participants with experience of psychosis alongside mental health professionals, dehumanisation researchers, and carers in a modified Delphi study. Items deemed most important were retained, and least important removed. There was an opportunity for research participants to suggest new items and amendments to existing items. Following the Delphi, research participants with experience of psychosis completed cognitive ‘think aloud’ interviews [24] with the DiPS. Items were reworded or removed if they caused confusion, offence, or were deemed as being too similar to other items on the measure.

#### Psychometric validation: item reduction and presentation

Research participants completed the DiPS alongside other measures of mental health for the purpose of psychometric evaluation and item reduction. Statistical properties of each item were considered to identify candidate items for removal. These items were presented to PPIE consultants in individual meetings, who each gave recommendations for whether they should be retained or removed. PPIE consultants also gave general feedback on the measure and its presentation.

#### Dissemination

PPIE consultants contributed through authorship of the present article to the dissemination of the DiPS and have contributed to the co-design of a lay summary and infographic. PPIE consultants have also advised on and been involved with dissemination strategies.

## Results

### Study document review

To ensure participant facing documents were acceptable and accessible to participants, a PPIE consultant reviewed the participant information sheet, consent form, recruitment poster, and debrief sheet for the development and validation studies. Consequently, useful feedback was given on how to ensure wording was sensitive, concise, and understandable.

### Identification of domains

Psychological theories describe self-dehumanisation as the denial of human nature and human uniqueness attributes [17] and mind [25] to the self. We considered it important to also include domains from qualitative research [23] on dehumanisation in people with lived experience of distressing voice-hearing, who described a broad range of experiences including the disintegration of ones sense of self, loss of trust in oneself, and distressing sensory fragmentation which have particular significance for self-dehumanisation in psychosis but were not described in previous dehumanisation literature.

These domains were discussed in a focus group by the DiPS lived experience consultation group. Group members explained how difficult it can be to reveal one’s true self to others, and how there is an ongoing feeling of difference and distance between self and others. They described not being free to live the life they want to live, and not always understanding experiences of psychosis, often questioning what is real. This questioning of reality and feelings would give rise to self-dehumanisation. When interacting with other people, there was a persistent, ongoing sense of meta-dehumanisation from police, mental health professionals, and society, while on the other hand, a humanising benefit of peer support. This was elaborated on by one member, who felt self-dehumanisation can be internalised after a lack of validation from others and feeling others see them as less human. PPIE consultants confirmed the validity of the themes generated in the qualitative study and literature review whilst highlighting important aspects of the dehumanisation experience. This confirmed that measure development was a useful avenue for research, and that we had a suitable starting point to build on.

### Item generation, refinement and amendment

A second PPIE focus group was convened in which members reviewed a list of items and generated additional items to include in the measure. PPIE consultants highlighted many important points that had been missed when drafting the items. The group emphasised the importance of including positively, as well as negatively phrased items, as the measure then in its present form was ‘devastating’. Group members spoke of the transience of both psychosis and feelings of dehumanisation. As a result of this refection and discussions between the research team, it was decided that the DiPS would contain a Likert scale asking its users to reflect on their experiences over the past week. Items were developed to capture the changing nature of psychosis, for example, replacing the item stem ‘my psychosis’ – which PPIE consultants felt sounded permanent and ‘like it belongs to somebody’, with ‘my experience of psychosis’. The group highlighted the importance of having the word ‘psychosis’ embedded in certain items, to give the reader clarity about context. PPIE consultants also suggested the measure considers that dehumanisation is experienced relationally, and often occurs as a result of interpersonal interactions and certain forms of treatment. This was an interesting point which led to a rich discussion. Psychological literature clearly demarcates self-dehumanisation and meta-dehumanisation as distinct processes [26], yet members of the PPIE group saw this differently and felt the two experiences were inseparable. Resultingly, we developed six items which captured the feeling of being dehumanised by certain members of society (“I feel I am not human when I am with…”, “the way I am treated by… makes me feel like I am not human”).

Content validity of the DiPS was further improved in subsequent Delphi and cognitive interview studies with research participants. In the Delphi, participant ratings determined which items were retained and removed, with an opportunity to suggest amendments embedded. Participant feedback and thoughts from the remaining items gathered in cognitive interviews was then used to improve the wording and further remove redundant items. This ensured all items were acceptable, relevant, and comprehensible.

### Psychometric validation: item reduction and presentation

PPIE consultant opinions for some items were mixed, and for others there was clear agreement. All those consulted felt the measure should be as short as possible whilst cohering with the original theoretical work, and should end on a positively phrased item. These consultations and the statistical analysis informed the item reduction to produce the final 13-item version of the DiPS.

### Dissemination

PPIE consultants emphasised the importance of dissemination. There was a recognition that a lot of work has been done in measure development, but it would all be for nothing if not shared widely. The current paper is co-authored with all members of the PPIE group. An infographic depicting the study findings and plain English summary have been co-designed with a PPIE consultant. PPIE consultants have suggested important areas for research dissemination, including to lived experience groups and NHS teams, as well how to present research findings in alternative formats, including infographics and videos. One PPIE consultant co-presented the study at an international research conference.

### Reflections on PPIE focus groups

Whilst the purpose of the meetings was to develop the DiPS, the group provided a space for members to reflect on experiences of psychosis and dehumanisation. For IC, this was immensely thought provoking, and powerful to hear the lived experiences of others. For ER, it was interesting to hear how their experiences of psychosis and dehumanisation differed to other members. Sharing experiences of dehumanisation with others also allowed AB to feel less isolated. EB was struck by hearing the extent to which members of the group had felt dehumanised by professionals and colleagues. Efforts were made to reduce power dynamics within the group and the collaborative process was emphasised. For AB, it felt unusual to speak with a person in a position of authority and feel their voice was being heard. The group was both a supportive and creative space whereby the measure really took shape – evolving from a list of domains into an initial set of items. Group members expressed feeling part of the research process and that their contributions were valued.

*I think during the focus groups it was like a moment of reflection because when you are discussing*,* I actually had to reflect about my life*,* how it is with psychosis*,* to be able to give points. So it was actually helping me a lot to be able to know. And also*,* learning from other people and realising that I’m not the only person who’s maybe experiencing what I’m experiencing. So*,* it actually really helps me*,* but I’m still left with*: Yes, I’ve given out my point of view, but what happens next? After the study, will I still just continue on being the way I am? How will it actually benefit me? – (LA)

### Reflections on working collaboratively

*As a person with lived experience of psychosis, being included in the research process has been therapeutic and humanising. A result of living with serious mental illness (SMI) can be that we lose a sense of agency and that things just happen to us, we become less important. Throughout my life I have felt powerless about the treatment I receive for psychosis, but to have my voice heard in a study which may inform how future treatments are developed for people like me has been healing. My experiences of psychosis and my opinions on the subject have felt valued, respected, and handled with psychological safety and dignity. Being amongst peers with similar experiences was validating and I felt less alone. Psychosis can be difficult to conceptualise if you’ve never experienced it, so it’s incredibly important we are included in research about it. This process embodies the disability rights slogan ‘nothing about us without us’. I’m so grateful I had the chance to be part of this study. It is a commendable example to future researchers*. – (AB)

*Working collaboratively with people with lived experience in developing the DiPS has been an invaluable experience and deeply enriched my own understanding. As a researcher without personal experience of psychosis, being able to learn first-hand from those who have has allowed me to become more aware of my blind spots, and more attuned to the impact psychosis can have. I was shocked and saddened to hear some of the stories shared in the focus groups and interviews, yet felt moved by those willing to use such difficult experiences to shape research and help improve the lives of others. Collaboration with the PPIE consultants brought the research to life for me in a way that no amount of reading could, and continues to motivate my work on this project*. – (TJ)

## Discussion

Across each stage of development, people with lived experience of psychosis made important contributions to the DiPS, both as PPIE consultants, and as research participants. All items contained within the final version of the measure have come from accounts of self-dehumanisation by people with lived experience of psychosis. Many of these have been suggested directly by people with lived experience, others have been amended based on lived experience feedback, and we have assurance that the measure is acceptable to those who complete it. Suggestions made on wording, both in the PPIE focus groups and cognitive interviews, align much with those by Connell and colleagues [27], who emphasised the importance of clear, meaningful, and sensitive items. The overall acceptability of the measure, as highlighted in the Delphi study and cognitive interviews highlights the benefit of involving qualitative inductive methods in measure development [28]. Including PPIE consultants in the final item reduction was felt to be an important step because model fit may well be at odds with lived experience priorities [29], and this approach allowed us to ensure the final version of the DiPS was both psychometrically sound and acceptable to PPIE consultants. Overall lived experience input has improved both content and face validity of the DiPS.

Alongside the improvement to research quality, we perceive benefits to both people with lived experience and researchers in the process of co-developing measures. Firstly, there is a longstanding history of people with psychosis not being listened to; many are dismissed as unreliable narrators of their experience [12], and all too often find that conversations with people in positions of power lead to bad news, including unwanted medication and detainment. Working collaboratively provides an opportunity to address the power imbalance inherent within research and clinical practice, enabling those with lived experience to have their voices heard, whilst improving the quality of the research being conducted. Secondly, involving PPIE consultants through focus groups played an important part in our measure development. Bringing people together to discuss aspects of their lived experience of psychosis is known to have therapeutic benefits, including universality [30]. PPIE consultants in focus groups reported valuing and connecting with each other through a rich and flowing discussion of their shared experiences of dehumanisation, and this supported the researcher’s aim to understand and operationalise experiences of dehumanisation. Therapeutic benefits to people with lived experience have also been reported in co-production of an intervention for obsessive-compulsive disorder (OCD) [31]. Third, ‘virtuous listening’ [32] and engagement in discussions may allow researchers without lived experience of a condition to improve their understanding through experiential learning [33].

### Critical perspective

Research participation and co-production are often wrongly conflated. However, our experiences with developing the DiPS have led us to recognise that research participation and co-production are also not simple, binary processes. Instead, they may better represent two ends of a spectrum of involvement. Participants in our Delphi study, for example, had an opportunity to suggest new items for the DiPS, and feedback from participants in cognitive interviews led to changes in item wording. Although this is a different distribution of power between researcher and participant to researcher and PPIE consultants, this is a different level of involvement to participants who took part in completing the questionnaire for psychometric validation, who had no opportunity for input into item content. As such, we recognise that methods of involvement such as Delphi and cognitive interviews can allow for meaningful lived experience input in measure development, whilst acknowledging that participation in this context does not qualify as true ‘co-production’.

Carlton and colleagues provide a gold standard framework for involving PPIE contributors throughout eleven stages of outcome measure development [15]. Our work on the DiPS meets many, but not all the recommendations made. Choice of self-dehumanisation in psychosis as a topic was not something directly suggested by people with psychosis; instead, it was an idea emergent from observations in clinical work [34]. PPIE focus group discussions, however, confirmed the importance of conducting research into self-dehumanisation, with the PPIE consultants feeding back that they felt this was an important and under-researched area to explore. Furthermore, PPIE consultants did not directly contribute towards the analysis of the data in either the qualitative interviews or cognitive interviews. However, qualitative themes were checked with 5 PPIE consultants, whose input was in validating themes generated by researchers, rather than coding themselves. Involving PPIE consultants in qualitative analysis is beneficial, as their perspective offers richness, and has been shown to deviate from researchers [35], thus broadening the interpretation of the data with potential benefits to overall measurement validity. Cognitive interviews could have been more collaborative and relied less on the researcher’s interpretation of participants thoughts. A more dialogical approach could allow perceptions to be more fully and clearly understood, with greater opportunity for clarifications to be made. There are clear advantages to following Carlton and colleague’s framework: more equal distribution of power, diversity of lived experience perspectives, ensuring that outcomes meaningful to service users are prioritised, and enhanced content validity of the measures. However, as stated by Grundy and colleagues [29], two challenges to working rigorously and collaboratively with multiple PPIE contributors on measure development are time and money. We were guided by the principles of the framework and made our best efforts for inclusion with the resources available.

### Recommendations

Based on experiences developing the DiPS, we offer some recommendations for those wishing to collaboratively develop measures:

- Lived experience involvement is essential to good quality measure development.
- Developing good quality measures takes time, but if done well, will only need to be done once. We recommend following gold standard methodology [14, 15, 20] to bring the most benefit to treatment evaluation and the development and testing of theoretical models.
- Attending to the wellbeing of PPIE consultants throughout the measure development process is essential. For example, when recruiting PPIE consultants it is useful to discuss materials and methods that may be triggering and agree how to collaboratively manage any distress. This is particularly important when the measure being developed relates to physical or mental health. We recommend a trauma-informed approach throughout, with a guiding principle being commitment to minimising discussion of potentially triggering or upsetting material.
- Researchers should acknowledge and recognise the value of all contributions, whilst being clear about what aspects of a measure can and can’t be changed.
- We recommend researchers plan and budget for lived experience involvement in advance of commencing project work, and seek additional funding throughout the research process if needed. In times where this is not possible and budgets are constrained, frameworks for involvement [14, 15] should be referenced as guides for activity, rather than prescriptive steps. To ensure people with lived experience are involved in generating research questions prior to receipt of grant funding, institutions may wish to invest in co-producing research strands from which ideas can be generated.
- Allow sufficient time within focus groups and individual interviews for PPIE consultants to share aspects of their histories and experiences that may not directly address research questions. Facilitators should enter their role with compassion and openness.
- Facilitators may wish to meet in-person or remotely with new PPIE contributors individually before any initial focus groups to establish rapport, and agree confidentiality, expectations, and boundaries of the focus group. This step was recommended by a PPIE consultant in the present study and was felt to be an important step for building trust, safety, and rapport.
- Clear communication between meetings is important; measure development is a lengthy process, and it can be helpful to share updates with those involved to keep people informed about progress.
- Consider benefits and drawbacks of meeting with PPIE contributors as a group v individually. We conducted individual interviews when making item reduction decisions, and focus group for the more creative stage of developing the initial pool of items and evaluating the broader theoretical framework. Individual interviews may help resolve finer details, whereas a focus group may facilitate broader creative discussions and sharing of experiences.
- Continuity of PPIE group members through stages of measure development is beneficial. The same five PPIE consultants worked on the DiPS throughout its development. This allowed the relationships built and ideas shared within the first focus group to flow into the second and any subsequent meetings.

### Conclusions

People with lived experience brought great value to the development and validation of the DiPS. We hope the DiPS will be a useful outcome measure in psychological research and therapy, enabling a focus on humanising care for people with psychosis. People with lived experience will be key to developing further understanding of dehumanisation.

## Data Availability

Not applicable.
